# Vitamin A supplementation status and associated factors among children aged 6–59 months in Tanzania: a multi-level analysis

**DOI:** 10.3389/fnut.2024.1422805

**Published:** 2024-08-06

**Authors:** Amanuel Yosef Gebrekidan, Yordanos Sisay Asgedom, Beshada Zerfu Woldegeorgis, Gizachew Ambaw Kassie, Kirubel Eshetu Haile, Solomon Abrha Damtew, Ashenafi Teklay, Amare Demsie Ayele

**Affiliations:** ^1^School of Public Health, College of Health Sciences and Medicine, Wolaita Sodo University, Wolaita Sodo, Ethiopia; ^2^Department of Internal Medicine, College of Health Sciences and Medicine, Wolaita Sodo University, Wolaita Sodo, Ethiopia; ^3^School of Nursing, College of Health Sciences and Medicine, Wolaita Sodo University, Wolaita Sodo, Ethiopia; ^4^Subuha Seasie Woreda Health Office, Edaga Hamus, Tigray, Ethiopia; ^5^Department of Pediatrics and Child Health Nursing, School of Nursing, College of Medicine and Health Sciences and Specialized Hospital, University of Gondar, Gondar, Ethiopia

**Keywords:** Tanzania, Tanzania Demographic and Health Survey, vitamin A supplementation (VAS), multi-level analysis, under five children

## Abstract

**Background:**

Vitamin A supplementation every 4–6 months is an economical, rapid, and effective strategy to enhance vitamin A status and minimize child morbidity and mortality due to vitamin A deficiency in the long run. Therefore, this study was aimed at investigating the level as well as the factors influencing VAS status among children aged 6–59 months in Tanzania.

**Methods:**

This analysis relied on data from the 2022 Tanzania Demographic and Health Survey (TDHS). The study used a weighted sample of 9,382 children aged 6–59 months. Given the effect of clustering and the binary character of the outcome variable, we employed a multilevel binary logistic regression model. The adjusted odds ratio (AOR) with a 95% confidence interval (CI) was used to determine statistical significance, considering the model with the lowest deviation that best fits the data.

**Results:**

In this study, vitamin A supplementation among children aged 6–59 months was found to be 53.18% [95% CI: 52.17, 54.19]. Mother’s/caregiver’s working status; Working [AOR = 1.59, 95% CI: 1.34, 1.89], ANC follow-up [AOR = 1.71, 95%CI: 1.34, 2.2], and health facility delivery [AOR = 1.55, 95%CI: 1.25, 1.91] were individual-level factors associated with vitamin A supplementation. Whereas administrative zones of Western [AOR = 2.02, 95% CI: 1.16, 3.52], Southern highlands [AOR = 3.83, 95% CI: 2.02, 7.24], Southern administrative zone [AOR = 2.69, 95% CI: 1.37, 5.3], and South West highlands [AOR = 0.56, 95% CI: 0.33, 0.95] were community-level factors associated with vitamin A supplementation.

**Conclusion:**

The proportion of VAS among children in Tanzania is low compared to UNICEF’s target of 80. Mother’s/caregiver’s working status, antenatal care, place of delivery, community-level media exposure, and administrative zones were significantly associated factors with vitamin A supplementation. Therefore, interventions should be designed to improve the uptake of VAS. Provision and promotion of ANC and institutional delivery and strengthening of routine supplementation are recommended to increase coverage of childhood vitamin A supplementation. Moreover, special focus should be given to regions in the south-western highlands.

## Introduction

Vitamin A is an essential nutrient for several physiological functions throughout all stages of life. Vitamin A and its derivatives regulate a wide range of functions, including reproduction, embryogenesis, vision, growth, cellular differentiation and proliferation, epithelial cellular integrity maintenance, and immunological function, as well as the development of children, and it is typically gained through a nutritious diet (1, 2). The primary animal sources of vitamin A are liver, eggs, milk, and milk products. Plant foods high in provitamin A account for more than 80% of total vitamin A intake due to their low cost, widespread availability, and diversity. Fruits, roots, tubers, and leafy vegetables are the primary sources of vitamin A carotenoids. Among fruits, mangoes are an important source of vitamin A. Yellow or orange-sweet potatoes are high in provitamin A. Red palm oil also contains high levels of provitamin A carotenoids (3). Retinol is the primary circulating form of vitamin A in the blood and plasma (4). Vitamin A (VA) improves immune function and decreases mortality from measles, diarrhea, and other infections (5).

Vitamin A deficiency occurs when dietary intake of vitamin A is insufficient to meet physiological requirements (4). Vitamin A deficiency is the major cause of preventable childhood blindness, increasing the risk of death from common childhood diseases, including measles and diarrhea. Conversely, vitamin A deficiency can also be aggravated by high infection rates, particularly diarrhea and measles. It is widespread in low-income countries and a public health concern, particularly in Africa and Southeast Asia, but uncommon in developed countries (2, 4).

Vitamin A deficiency affects around 190 million preschool-aged children, the majority of whom live in Africa and Southeast Asia, and there is evidence linking it to severe acute malnutrition. If left untreated, vitamin A deficiency can lead to blindness, an increased risk of disease, and mortality from childhood infections (6). In 2013, the prevalence of vitamin A deficiency, considering data from 83 countries across the globe, was found to be 29% (7). Between 1991 and 2013, vitamin A deficiency decreased from 42 to 6% in East and Southeast Asia and Oceania, as well as from 21 to 11% in Latin America and the Caribbean. In 2013, Sub-Saharan Africa had the highest prevalence of vitamin A deficiency (48%), followed by South Asia (44%) (7). Vitamin A deficiency caused 94,500 fatalities from diarrhea and 11,200 deaths from measles, accounting for 1.7 percent of all deaths in children under 5 years old in low-and middle-income countries. More than 95% of the deaths occurred in sub-Saharan Africa and South Asia (7).

Different studies conducted in Tanzania have shown a varying degree of vitamin A deficiency. A study conducted to determine the effect of vitamin A deficiency on the anthropometric attributes of children in the Manyara and Shinyanga regions of Tanzania found that the prevalence of vitamin A deficiency among children aged 6–36 months was 69.5% (8). Another study conducted in Kikwawila and Kiberege wards, Tanzania, on primary schoolchildren aged 6–12 years showed that the magnitude of vitamin A deficiency was 34.71% (9).

On April 1, 2016, the United Nations (UN) General Assembly proclaimed 2016–2025 the United Nations Decade of Action on Nutrition. Led by World Health Organization and the Food and Agriculture Organization of the United Nations (FAO), the UN Decade of Action on Nutrition calls for policy action across six key areas, including aligning health systems to nutrition needs and providing universal coverage of essential nutrition interventions (10).

Vitamin A supplementation twice a year is linked to a clinically relevant reduction in morbidity, and it was found to reduce the risk of all-cause death by 12% when compared to the non-supplemented controls. Additionally, it will lead to a 12% reduction in deaths caused by diarrhea, a 15% reduction in the incidence of diarrhea, and a 50% reduction in the incidence of measles (7, 11). Therefore, supplementation with vitamin A every 4–6 months is an economical, rapid, and effective strategy to enhance vitamin A status and minimize child morbidity and mortality in the long run (2, 12). In many countries, vitamin A supplementation for newborns and children has been successfully combined with routine immunization programs and vaccination campaigns. Furthermore, delivering high-dose supplements to mothers at vaccination centers soon after birth provides an additional benefit to young infants through enriched breast milk (2).

The coverage of vitamin A supplementation for children aged 6–59 months rose from 30% in 2008–2009 to 72% in 2014 worldwide (13). Even though the importance of vitamin A supplementation is proven, the coverage and utilization of vitamin A supplementation are still below target in most countries. A United Nations International Children’s Emergency Fund (UNICEF) report in 2018 shows that in 2016, only 64% of children in need in priority countries received two doses of vitamin A; yet, more than 141 million children were left behind, making them vulnerable to disease and death (13). According to data from Nutrition International, 167 million under-five children received two doses of vitamin A in 2021, and 80% of those who need vitamin A supplements in developing countries were reached (11). The 2016 Tanzania Demographic and Health Survey (TDHS) and the 2018 Tanzania nutrition survey showed that the coverage of vitamin A supplementation among 6–59 month-old children was 41 and 63.8%, respectively (14, 15). A UNICEF report in 2022 indicates a two doses of vitamin A coverage was 90% in Tanzania, which indicates a 6 % reduction compared to 96% coverage in 2021 (16). Despite this, previous studies have shown that the national VAS coverage is based on a tally-sheet system, which can estimate vitamin A supplementation 30% higher than the actual coverage. Additionally, there are factors, including health worker measurement errors in summarizing the count sheets and underestimating target population statistics, that indicate the need for nation-wide representative studies (17).

Different studies associate different factors with the uptake of vitamin A supplementation, both at the individual level and at the community level. Age of the mother, educational status of the mother, mother’s occupation, and household wealth status were frequently mentioned as individual-level sociodemographic factors (18–21). Maternal obstetric factors such as antenatal care, place of delivery, and postnatal care were found to be significantly associated with vitamin A supplementation (18, 20, 21). The age of the child is described as a child-related characteristic associated with vitamin A supplementation (18–20). Region (area) and residence were found to be factors associated with vitamin A supplementation at a community level (18–20).

Although there is evidence on the level of VAS in Tanzania, the majority of the evidence is derived from government reports, and available studies either utilized data from the previous demographic and health surveys conducted or were conducted on a small scale and cannot represent the whole country, with an additional lack to identify national-level factors.

Therefore, this study was aimed at using the most recent 2022 Tanzania Demographic and Health Survey and Malaria Indicator Survey (2022 TDHS-MIS) data to investigate the level as well as the factors influencing VAS status among children aged 6–59 months in Tanzania, aiming to provide current information on the national-level VAS and its determinants, facilitating evidence-based decision-making.

## Materials and methods

### Study design and study area

We conducted a secondary data analysis study by using cross-sectional designed data from the 2022 Tanzania Demographic and Health Survey and Malaria Indicator Survey (2022 TDHS-MIS). Tanzania is organized into 31 regions; 26 in mainland Tanzania and 5 in Zanzibar (22). According to the 2022 population and housing census of Tanzania, the country has a total of 61.7 million population of which 59,851,347 reside in mainland Tanzania while 1,889,773 reside in Zanzibar. Of the total population, 40.2 million and 21.5 million people reside in rural and urban areas, respectively. Out of the total population, 31.7 million were females while the remaining 30 million were males (23). The 2022 Tanzania Demographic and Health Survey and Malaria Indicator Survey (2022 TDHS-MIS) is the seventh in a series of Demographic and Health Surveys (DHS) in Tanzania, carried out through the DHS Program. Previous DHSs were conducted in 1991–1921, 1996, 1999, 2004–2005, 2010, and 2015–2016. The 2022 TDHS-MIS aims to give current and credible information on population and health matters (22). To achieve this, a nationally representative sample of 16,354 households were chosen for the survey (22). Tanzania was divided into nine zones to evaluate spatial differences in population indicators. The Reproductive and Child Health Section of the Ministry of Health uses this classification scheme, despite the fact that these zones are not formal administrative areas. Grouping regions into zones results in larger denominators and smaller sampling errors for indicators at the zonal level (22).

### Data source, population and sampling procedure

This study was based on the recent 2022 TDHS-MIS, which is conducted to update health and health-related indicators. The 2022 TDHS-MIS sample design involved two stages and included estimates for the entire country, urban and rural areas in Tanzania Mainland, and Zanzibar (22). The first stage involved selecting sampling points (clusters) from enumeration areas (EAs) identified for the 2012 Tanzania Population and Housing Census (2012 PHC). EAs were chosen with a probability proportional to their size within each sampling stratum. A total of 629 clusters were identified. Of the 629 EAs, 211 were from urban areas and 418 from rural areas (22). The second stage involved selecting 26 households from each cluster, resulting in a total sample size of 16,354 for the 2022 TDHS-MIS. Prior to the main survey, all selected EAs underwent household listing operations, and households were selected from each cluster using a systematic sampling (22).

This study used data from interviewed women with under-five children (TZKR data). The investigation comprised 9,382 weighted sample children aged 6–59 months.

### Study variables and measurement

This study’s dependent variable was vitamin A supplementation among children aged 6–59 months within the previous 6 months before the survey. It was assessed by reviewing the integrated child health card, which includes immunization and growth monitoring information, as well as the mother’s spoken response, to determine if they have received vitamin A supplementation within the past 6 months. Because of the hierarchical nature of the DHS data, we evaluated individual and community-level variables as independent variables. Individual-level variables such as sociodemographic and economic variables, child-related characteristics, and a mother’s/caregiver’s obstetric characteristics were considered individual-level independent variables. The sociodemographic and economic variables included were the mother’s/caregiver’s age, the sex of the household head, marital status, mother’s/caregiver’s educational level, husband’s/partner’s educational level, mother’s/caregiver’s occupation, husband’s/partner’s occupation, and wealth index. The wealth index of households receives scores based on the number and types of consumer goods they own, including televisions, bicycles, or cars, as well as dwelling qualities like sources of drinking water, bathroom facilities, and flooring materials. These scores are calculated using principal component analysis. National wealth quintiles are calculated by assigning a household score to each household member, ranking each person based on their score, and splitting the distribution into five equal categories, each representing 20% of the population. It was categorized as poorest, poorer, middle, richer, and richest (22).

Independent variables under the child-related characteristics include the child’s age and the sex of the child, whereas independent variables under mother’s/caregiver’s obstetric characteristics include parity; parity was categorized as low parity if the mother or caregiver has never given birth or gave birth once; multiparous if the mother or caregiver gave two up to four births; and grand multiparous if the mother or caregiver gave five or more births. Antenatal care (ANC) was assessed if a woman with a live birth or a stillbirth in the last 2 years received antenatal care from a skilled provider for the most recent birth (24). The place of delivery of the child was categorized as home if the child was delivered at places other than a health facility and as a health facility if the child was delivered at a health facility (25); and number of under-five children in the household, were used as independent variables. The community-level variables were residence, administrative zones and community-level media exposure. Administrative zones: Tanzania was divided into nine zones to evaluate spatial differences in population indicators. The Reproductive and Child Health Section of the Ministry of Health uses this classification scheme, despite the fact that these zones are not formal administrative areas. Grouping regions into zones results in larger denominators and smaller sampling errors for indicators at the zonal level. The administrative zones were classified as main land Tanzania (8 zones): Western zone: Tabora, Kigoma; Northern zone: Kilimanjaro, Tanga, Arusha; Central zone: Dodoma, Singida, Manyara; Southern Highlands zone: Iringa, Njombe, Ruvuma; Southern zone: Lindi, Mtwara; Southwest Highlands zone: Mbeya, Rukwa, Katavi, Songwe; Lake zone: Kagera, Mwanza, Geita, Mara, Simiyu, Shinyanga; Eastern zone: Dar es Salaam, Pwani, Morogoro; and Zanzibar (1 zone): Zanzibar zone: Kaskazini Unguja, Kusini Unguja, Mjini Magharibi, Kaskazini Pemba, Kusini Pemba (22). Community media exposure was classified as “yes” if they had access to all three media (read a newsletter, listen to a radio, and watch television) at least once a week, and “no” if they had no media exposure (25).

### Data processing and analysis

The data were analyzed in Stata version 14 (StataCorp, United States) following the eligibility check. A multilevel analysis was carried out. The model’s eligibility was determined by calculating the Intra-class Correlation Coefficient (ICC), and a model with ICC more than 10% was selected for multilevel analysis. In this study, the ICC was 18.2%. Because the data were hierarchical (individuals were nested within communities), a two-level mixed-effects logistic regression model was fitted to estimate both the individual and community level variables (fixed and random effect) on vitamin A supplementation status, and the log of the probability of VAS was modeled using the formula below (26):


$$\log\left[\frac{\Pi_{ij}}{1-\Pi_{ij}}\right]=\beta_{0}+\beta_{1}X_{ij}+\beta_{2}Z_{ij}+\mu_{j}+e_{ij}$$

In this equation, *i* and *j* represent individual and community units, while *X* and *Z* represent individual and community-level variables, respectively. Π_ij_ represents the probability of vitamin A supplementation for the ith 6–59 months aged child in the jth community. The (β) will be the fixed coefficients. The intercept (β_0_) represents the probability of Vitamin A supplementation in the absence of predictors; the random effect (μ_j_) represents the effect of the community on VAS for the jth community, whereas eij represents random errors at the individual level. Assuming each community has a unique intercept (β_0_) and fixed coefficient (β), we can account for clustered data and variances between communities (26).

After being weighted with sampling weight, primary sampling unit, and strata to account for the sampling design and restore survey representativeness. Because the outcome variable was binary, we used a multilevel binary logistic regression model to investigate the associated factors of Vitamin A supplementation. Because DHS data are hierarchical, under five children are nested inside clusters, and subjects within the same cluster may have comparable characteristics with those in another cluster, so breaching the independence and equal variance criteria. As a result, an advanced multilevel cumulative logit model was applied to account for cluster heterogeneity. To quantify the extent of cluster variation in Vitamin A supplementation status, four models for multilevel binary logistic regression analysis were developed. These models included a null model with no explanatory factors. The second model was modified for individual-level factors, and the third for community-level variables. The fourth model was fitted simultaneously with variables at the individual and community levels. To find the best-fitted model for the data, model fitness was assessed using deviance [−2Log-Likelihood Ratio (LLR)], with the model with the lowest deviation being the most appropriate. In the bivariable multilevel binary logistic regression model, variables having a *p*-value ≤0.2 were considered for the multivariable analysis. The multivariable analysis gave an adjusted odds ratio (AOR) with a 95% confidence interval (CI). Variables with a p-value <0.05 were identified as significantly associated factors.

Measures of variation (random-effects) were reported using ICC, median odds ratio (MOR), and proportionate change in variance (PCV) to compare variations between clusters. The ICC measures the similarity of observations within a cluster, while MOR assesses unexplained cluster heterogeneity (26). MOR is defined as the median odds ratio between the highest and lowest risk areas when two areas are chosen at random (26). In this study, MOR demonstrates how much the residential cluster influences an individual’s likelihood of vitamin A supplementation. The proportionate change in variance (PCV) calculates the overall variation caused by both individual-level and area-level components in a multilevel model (26).

### Ethical consideration

Our study did not require ethical approval or participant agreement because it was a secondary analysis of publicly available survey data from the MEASURE DHS program. However, the 2022 TDHS-MIS questionnaires and survey protocol were approved and authorized by the Medical Research Council of Tanzania and the Zanzibar Health Research Institute. The ICF’s Internal Review Board also reviewed the study. The dataset was retrieved from the Demographic and Health Survey program website^1^ after receiving consent to use it. Furthermore, the dataset did not include any personally identifiable information, such as names or household numbers (identifiers).

## Results

### Socio-demographic characteristics of respondents

The study included a weighted sample of 9,382 mothers/caregivers with children aged 6–59 months (Table 1). The mean age of the mothers was 27.79 (SD ± 7.04) years, with the majority (78.83%) of the household heads being male. Nearly one-third (32.39%) of mothers are not working, and 22.85% of households fall into the poor wealth index (Table 1).

**Table 1:** Sociodemographic and economic characteristics of study participants who have children aged 6–59 months in Tanzania.

Variable	Category	Weighted frequency	Percent
Mother’s/caregiver’s age (in years)	15–19	396	4.22
	20–24	2,225	23.72
	25–29	2,511	26.76
	30–34	1843	19.65
	35–39	1,443	15.38
	40–44	740	7.89
	45–49	224	2.39
Sex of the house hold head	Male	7,395	78.83
	Female	1987	21.17
Marital status	Never in a union	574	6.1
	Married	5,521	58.8
	Living with partner	2,278	24.2
	Widowed	117	1.2
	Divorced	544	5.8
	Separated/no longer living together	348	3.7
Mother’s/Caregiver’s education level	No education	2016	21.49
	Primary	5,349	57.01
	Secondary	1922	20.49
	Higher	95	1.01
Husband’s/partner’s education level (*n* = 7,799)	No education	1,173	15.04
	Primary	4,745	60.84
	Secondary	1,636	20.98
	Higher	245	3.14
Mother’s occupation	Not working	3,039	32.39
	Professional/technical/managerial	238	2.54
	Clerical	22	0.23
	Sales	805	8.58
	Agricultural – employee	2,227	23.74
	Services	18	0.19
	Skilled manual	1,050	11.19
	Unskilled manual	1,558	16.61
	Other	425	4.53
Husband’s/partner’s occupation (*n* = 7,799)	Not working	700	8.98
	Professional/technical/managerial	668	8.75
	Clerical	18	0.22
	Sales	448	5.74
	Agricultural – employee	2,344	30.06
	Services	130	1.66
	Skilled manual	1,542	19.78
	Unskilled manual	1,586	20.34
	Other	363	4.66
Wealth index	Poorest	2,144	22.85
	Poorer	1856	19.78
	Middle	1779	18.97
	Richer	1901	20.26
	Richest	1702	18.14

### Mothers/caregivers obstetrics related characteristics

Out of the total 9,382 included mothers/caregivers, 55.29% are multiparous, while 29.54% are grand multiparous. Out of 4,612 mothers who were asked for antenatal care follow-up, 89.4 had ANC follow-up, and the majority, 81.2%, delivered their last child in a health facility. More than half, or 52.48%, of the households have two to three under five children in their houses (Table 2).

**Table 2:** Obstetrics-related characteristics of respondent mothers/caregivers who have children aged 6–59 months in Tanzania.

Variable	Category	Weighted frequency	Percent
Parity	Low parity	1,423	15.17
	Multiparous	5,187	55.29
	Grand multiparous	2,772	29.54
Antenatal care (ANC) (4612)	Yes	4,123	89.4
	No	499	10.6
Number of ANC follow up (4612)	No visits	489	10.6
	One visit	70	1.51
	Two visits	238	5.17
	Three visits	776	216.82
	Four visits and above	3,039	65.9
Place of delivery (5262)	Home	989	18.8
	Health facility	4,273	81.2
Number of under 5 children	0–1	3,824	40.76
	2–3	4,924	52.48
	More than 4	634	6.76

### Child characteristics

The mean age of the children was 32.07 (standard deviation ±15.69) months, with 50.88% being male (Table 3).

**Table 3:** Child-related characteristics among study participants aged 6–59-month-old children in Tanzania.

Variable	Category	Weighted frequency	Percent
Age of the child (in months)	6–11	1,073	11.43
	12–23	2,180	23.24
	24–35	2009	21.42
	36–47	2023	21.56
	48–59	2097	22.35
Sex of the child	Male	4,774	50.88
	Female	4,608	49.12

### Community level characteristics

The majority of the study participants (72.63%) reside in urban areas, with more than one-third (34.22%) living in the Lake Administrative Zone. More than half of the households (59.85%) have no media exposure (Table 4).

**Table 4:** Community-level characteristics of study participants who have children aged 6–59 months in Tanzania.

Variable	Category	Weighted frequency	Percent
Residence	Urban	2,568	27.37
	Rural	6,814	72.63
Administrative zones	Western	962	10.25
	Northern	1,024	10.92
	Central	963	10.26
	Southern highlands	472	5.03
	Southern	344	3.66
	South west highlands	881	9.39
	Lake	3,211	34.22
	Eastern	1,242	13.24
	Zanzibar	283	3.02
Community level media exposure	Yes	3,767	40.15
	No	5,615	59.85

### Vitamin A supplementation uptake among children aged 6–59 months in Tanzania

**Figure 1:**
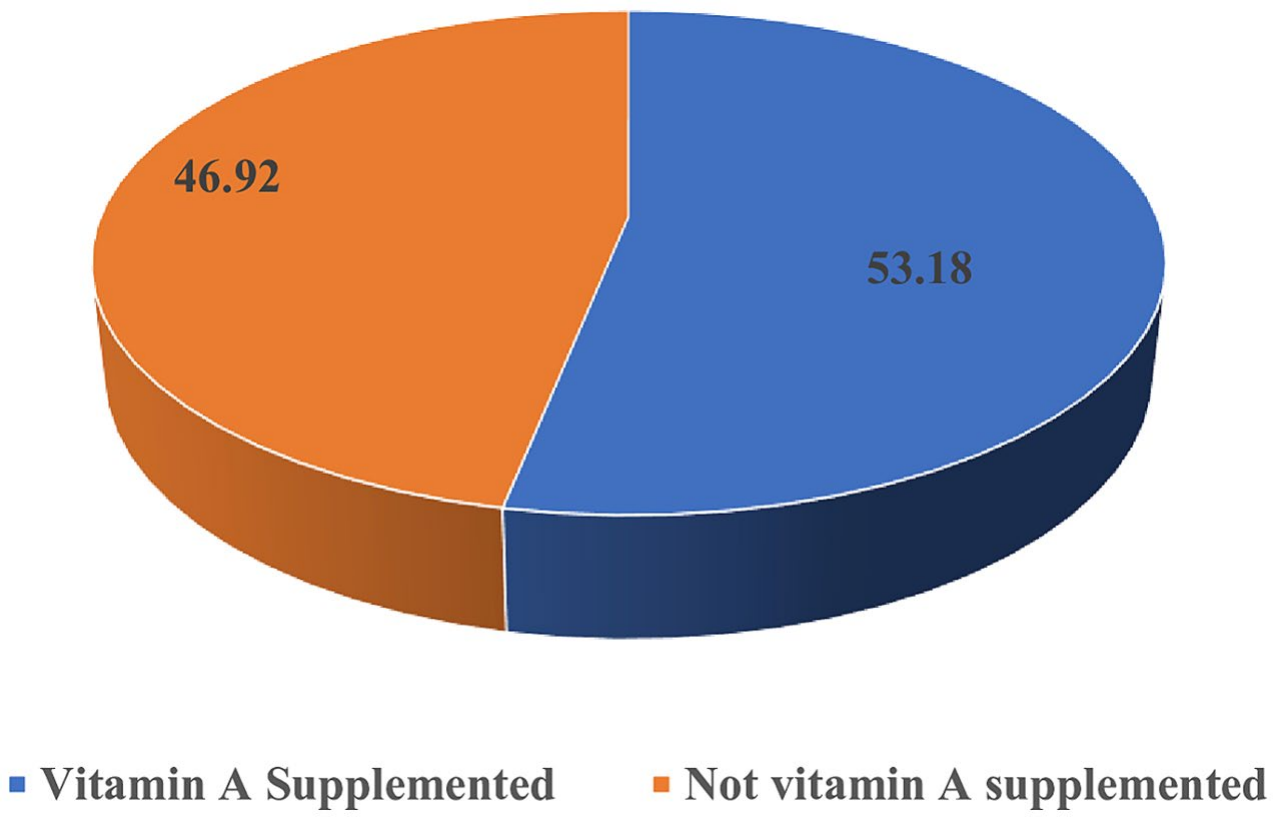
Proportion of vitamin A supplementation status among 6–59-month-old children in Tanzania.

The overall uptake of vitamin A supplementation in Tanzania among children aged 6–59 months in the last 6 months prior to the survey was 53.18% [95% CI: 52.17, 54.19] (Figure 1).

### Random effect analysis (measures of variation)

#### Null model (model 1)

This model is intercept-only, with no predictors. We used the Likelihood Ratio to determine if the multilevel binary logistic regression model was more significant than the single-level binary logistic regression model. The LR test findings were statistically significant (*p* < 0.01), showing that the multilevel binary logistic regression model best fit the single-level binary logistic regression analysis. As a result of the LR test, a multilevel binary logistic regression model was used to identify factors associated with vitamin A supplementation. Four random effects models were fitted, and the final model was chosen based on the lowest deviation value.

The null model’s ICC was 18.12%, indicating that 18.12% of the overall variability in Vitamin A supplementation uptake was due to cluster variation. Furthermore, the median odds ratio (MOR) for vitamin A supplementation uptake was 1.92, indicating variance between clusters. Those children aged 6–59 months in the cluster with higher vitamin A supplementation status had a 1.92 times higher likelihood of vitamin A supplementation than those in the cluster with lower vitamin A supplementation status. The best-fit model was selected based on the lowest deviation value (4751.16) (Table 5).

**Table 5:** Multilevel analysis of factors associated with vitamin A supplementation uptake among children aged 6–59 months in Tanzania.

Variables	Received vitamin A supplementation in the last 6 months		COR (95% CI)	Model 1 AOR (95% CI) (With level one variables)	Model 2 AOR (95% CI) (With level two variables)	Model 3 AOR (95% CI) (With level one and two variables)
	Yes *n* (%)	No *n* (%)				
Individual level variables						
Mother’s/caregiver’s age (in years)						
15–19	221 (55.83)	175 (44.17)	1.63 (1.13, 2.34)	1.07 (0.55, 2.12)		1.1 (0.56, 2.17)
20–24	1,147 (51.53)	1,079 (48.47)	1.3 (0.96, 1.76)	0.81 (0.45, 1.47)		0.8 (0.44, 1.44)
25–29	1,427 (56.82)	1,084 (43.18)	1.64 (1.21, 2.22)	1.03 (0.58, 1.84)		0.99 (0.56, 1.77)
30–34	952 (51.63)	892 (48.37)	1.33 (0.98, 1.81)	0.91 (0.52, 1.6)		0.89 (0.51, 1.56)
35–39	738 (51.19)	704 (48.81)	1.21 (0.88, 1.65)	0.94 (0.53, 1.64)		0.92 (0.53, 1.62)
40–44	402 (54.37)	338 (45.63)	1.46 (1.05, 2.03)	0.93 (0.11, 1.68)		0.91 (0.5, 1.65)
45–49	102 (45.62)	122 (54.38)	1	1		1
Mother’s educational status						
No education	904(44.86)	1,112 (55.14)	1	1		1
Primary education	2,901 (54.24)	2,448 (45.76)	1.27 (1.12, 1.43)	1.19 (0.97, 1.46)		1.18 (0.97, 1.45)
Secondary education	1,131 (58.82)	791 (41.18)	1.47 (1.26, 1.71)	1.1 (0.83, 1.45)		1.1 (0.83, 1.46)
Higher education	53 (55.88)	42 (44.12)	1.31 (0.82, 2.08)	1.28 (0.5, 3.03)		1.33 (0.52, 3.4)
Husband/partners educational level (*n* = 7,799)						
No education	518 (44.17)	655 (55.83)	1	1		1
Primary education	2,531 (53.34)	2,214 (46.66)	1.16 (1.01, 1.35)	1.1 (0.87, 1.39)		1.06 (0.84, 1.34)
Secondary education	940 (57.44)	696 (42.56)	1.38 (1.15, 1.65)	1.26 (0.94,1.69)		1.25 (0.93, 1.68)
Higher education	141 (57.69)	104(42.31)	1.44 (1.04, 1.99)	1.65 (0.96, 2.83)		1.66 (0.96, 2.84)
Mother’s occupation						
Not working	1,442 (47.46)	1,597 (52.54)	1	1		1
Working	35.47 (55.92)	2,796 (44.08)	1.51 (1.37, 1.67)	1.59 (1.34, 1.89)		1.59 (1.34, 1.89) ***
Husband’s/partner’s occupation (*n* = 7,799)						
Not working	304 (43.49)	396 (56.51)	1	1		1
Not working	3,825 (53.89)	3,274 (46.11)	1.39 (1.17, 1.67)	0.95 (0.71, 1.27)		0.95 (0.72, 1.27)
Wealth index						
Poorest	933 (43.54)	1,211 (56.46)	1	1		1
Poorer	979 (52.77)	877 (47.23)	1.29 (1.11, 1.49)	1.28 (1.01, 1.62)		1.25 (0.99, 1.58)
Middle	985(55.38)	794 (44.62)	1.41 (1.21, 1.65)	1.16 (0.9, 1.5)		1.11 (0.86, 1.44)
Richer	1,095 (57.59)	806 (42.41)	1.62 (1.37, 1.92)	1.28 (0.97, 1.68)		1.18 (0.89, 1.56)
Richest	996 (58.51)	706 (41.49)	1.86 (1.54, 2.23)	1.42 (1.04, 1.95)		1.26 (0.91, 1.76)
Parity						
Low parity	891 (62.63)	532 (37.37)	1.63 (1.41, 1.89)	1.06 (0.74, 1.51)		1.06 (0.74, 1.81)
Multiparous	2,768 (53.13)	2,419 (46.63)	1.19 (1.07, 1.32)	1.16 (0.92, 1.46)		1.14 (0.9, 1.43)
Grand multiparous	1,330 (47.97)	1,442 (52.03)	1	1		1
Antenatal care (ANC) (*n* = 4,612)						
Yes	2,465 (59.79)	1,658 (40.21)	1.94 (1.55, 2.41)	1.69 (1.32, 2.17)		1.71 (1.34, 2.21) ***
No	222 (45.44)	267 (54.56)	1	1		1
Place of delivery (*n* = 5,262)						
Home	434 (43.93)	555 (56.07)	1	1		1
Health facility	2,565 (60.03)	1708 (39.97)	1.7 (1.44, 2.01)	1.67 (1.35, 2.06)		1.55 (1.25, 1.91) ***
Number of under five children						
1	2,283 (59.69)	1,541 (40.31)	1.44 (1.44, 2.01)	1.29 (0.91, 1.83)		1.36 (0.95, 1.94)
2–3	2,419 (49.12)	2,504 (50.88)	1.03 (0.84, 1.25)	1.02(0.73, 1.43)		1.11 (0.79, 1.56)
4–5	288 (45.36)	347 (54.64)	1	1		1
Community level variables						
Administrative zones						
Western	541 (56.29)	421 (43.71)	1.12 (0.75, 1.66)		1.18 (0.79, 1.77)	2.02 (1.16, 3.52) *
Northern	530 (51.74)	494 (48.26)	0.85 (0.58, 1.23)		0.87 (0.6, 1.27)	1.18 (0.7, 1.99)
Central	461 (47.87)	502 (52.13)	0.69 (0.47, 0.99)		0.72 (0.49, 1.05)	0.81 (0.48, 1.37)
Southern highlands	392 (82.99)	80 (17.01)	4.17 (2.69, 6.46)		4.2 (2.72, 6.51)	3.83 (2.02, 67.24) ***
Southern	246 (71.57)	98 (28.43)	1.21 (1.11, 3.02)		2.05 (1.29, 3.24)	2.69 (1.37, 5.3) **
South West highlands	381 (43.22)	500 (56.78)	0.55 (0.38, 0.8)		0.56 (0.39, 0.81)	0.56 (0.33, 0.95) *
Lake	1,555(48.42)	1,656 (51.58)	0.64 (0.46, 0.9)		0.66 (0.47, 0.93)	1.1 (0.69, 1.77)
Eastern	725 (58.4)	517 (41.6)	1.14 (0.79, 1.64)		1.15 (0.8, 1.66)	1.4 (0.85, 2.32)
Zanzibar	158 (55.96)	125 (44.04)	1		1	1
Community level media exposure						
Yes	2,124 (56.38)	1,643 (43.62)	1.2 (1.09, 1.33)		1.21 (1.09, 1.33)	1.18 (0.99,1.41)
No	2,865 (51.03)	2,750 (48.97)	1		1	
Measures of variation						
Variance	0.73		0.58		0.47	0.47
ICC	0.1812		0.1499		0.1259	0.1245
AIC	12191.71		4888.94		12139.2	4821.16
BIC	12205.99		5051.29		12217.71	5039.7
PCV	Ref.		20.54%		35.62%	35.62%
MOR 1.92 [95% CI: 1.72, 2.12]						
Model fitness (Goodness of fit)						
LLR	−6093.86		−2418.47		−6058.6	−2375.58
Deviance (−2LLR)	12187.72		4836.94		12117.2	4751.66
LR-test	654.91		141.86		493.66	113.64

*P-value < 0.05, **P-value < 0.01. ***P-value < 0.001. AIC, Akaike Information Criteria; AOR, Adjusted Odds Ratio; BIC, Bayesian Information Criteria; ICC, Intra-class Correlation Coefficient; LLR, Log-Likelihood Ratio; LR, Likelihood Ratio; MOR, Median Odds Ratio; PCV, Proportionate Change in Variance.

#### Factors associated with vitamin A supplementation among children aged 6–59 months in Tanzania

Bivariable analysis was used to identify candidate variables for the multilevel multivariable mixed-effect binary logistic regression. As a result, mother’s/caregivers age, mother’s/caregiver’s educational status, husband’s educational status, mother’s/caregiver’s occupation, husband’s/partner’s occupation, wealth index, parity, ANC, place of delivery, number of under-five children, administrative zones, and community-level media exposure were associated with vitamin A supplementation. In the multilevel multivariable mixed effect binary logistic regression model, mother’s/caregiver’s occupation, ANC, place of delivery, and administrative zones were found to be statistically significant factors associated with vitamin A supplementation among 6–59-month-old children in Tanzania. The odds of having vitamin A supplementation among children with mother’s/caregiver’s who were working (employed) were found to be 1.59 times [AOR = 1.59, 95% CI: 1.34, 1.89], higher compared to those children with mother’s/caregiver’s who were not working. Children with mother’s/caregiver’s who had ANC follow-up were 1.71 times more likely [AOR = 1.71, 95%CI: 1.34, 2.22] to be vitamin A supplemented than those children with mother’s/caregiver’s who had no ANC follow-up. Children who were born in health facilities had 1.55 times more odds [AOR = 1.55, 95% CI: 1.25, 1.91] to be vitamin A supplemented than those children who were delivered at home. Regarding administrative zones, children living in Western, Southern highlands, and Southern administrative zones were 2.02 times [AOR = 2.02, 95% CI: 1.16, 3.52], 3.83 times [AOR = 3.83, 95% CI: 2.02, 7.24], and 2.69 times [AOR = 2.69, 95% CI: 1.37, 5.3] more likely to be vitamin A supplemented than children living in Zanzibar. Meanwhile, the odds of vitamin A supplementation in children living in the South West highlands were 44% less compared to children Zanzibar (Table 5).

## Discussion

This study aimed at using the most recent 2022 Tanzania Demographic and Health Survey and Malaria Indicator Survey (2022 TDHS-MIS) data to investigate the level as well as the factors influencing VAS uptake among children aged 6–59 months in Tanzania. The study showed that only 53.18% [95% CI: 52.17, 54.19] of children aged 6–59 months had taken vitamin A supplementation in Tanzania. After adjusting for individual and community-level factors, mother’s/caregiver’s occupation, ANC, place of delivery of the current child, administrative zones, and community-level media exposure were significantly associated with vitamin A supplementation among children aged 6–59 months.

In this study, 53.18% of children have taken VAS, this finding is higher than studies conducted in Ethiopia among children aged 6–23 months reporting a vitamin A supplementation status of 47.2 and 43.4% (18, 25) and a study conducted in Guinea with a reported VAS status of 42% among children aged 6–59 months (27). This might be due to the difference in study population, in which this study considered children aged 6–59 months while the later studies took children aged 6–23 months. To the contrary, this study showed the status of vitamin A supplementation in Tanzania is below UNICEF’s target of 80%, a UNICEF report in 2022, which indicates a two-dose vitamin A supplementation coverage of 90% in Tanzania, and Tanzania’s 2018 nutrition survey report of 63.8% (13, 15, 16). These discrepancies might be attributed to the national VAS coverage based on a tally-sheet system, which can estimate vitamin A supplementation 30% higher than the actual coverage. Recent government data significantly overestimates VAS coverage. The huge difference in coverage percentages could be attributed to a number of factors, including health worker measurement errors in summarizing the count sheets and underestimating target population statistics (17, 28). It underscores the necessity to conduct representative population-based coverage surveys to supplement and validate tally-sheet estimations.

It was also found to be lower compared to a study conducted by including 23 Sub-Saharan countries that reported 59.4% vitamin A supplementation status (19). The possible explanation for this finding might be the difference in sociodemographic and economic status between these countries. This finding indicates Tanzania is lagging behind UNICEF’s target and the average of its fellow sub-Saharan countries, emphasizing the amount of work needed to reach the 80% target. Moreover, the finding of this study also revealed that the prevalence of VAS in Tanzania is lower than the reported prevalences of VAS in studies conducted in India and Pakistan among children aged 6–59, with reported nation coverages of 60.5 and 68.5%, respectively. The possible explanation for this difference could be the socioeconomic differences between the countries (29, 30).

This study identified significant individual and community-level factors that are associated with vitamin A supplementation. The odds of vitamin A supplementation among children aged 6–59 months with mother’s/caregiver’s who were working (employed) was found to be higher compared to those 6–59-month-old children with mother’s/caregiver’s who were not working. This finding is supported by a study conducted in Ethiopia where micronutrient uptake was found to be higher among children with mothers who had work compared to those with mothers who were not working, and a study about vitamin A supplementation among preschool children in Ethiopia (20, 25). The finding was also in line with studies conducted in Nigeria, Guinea and a multilevel analysis of 20 sub-Saharan African countries, where children with working mothers were found to be more likely to receive VAS than those without work (19, 27, 31). This might be explained as working mothers may have greater access to information about the benefits of VAS from colleagues at work or through community mobilization activities (20, 27).

Additionally, children with mother’s/caregiver’s who had ANC follow-up were more likely to be vitamin A supplemented than those children with mother’s/caregiver’s who had no ANC follow-up. This is also reported in studies conducted in Ethiopia (18, 20, 25, 32) and Nigeria (21). Moreover, children aged 6–59 months who were born in health facilities had a greater odd of being vitamin A supplemented than those children aged 6–59 months who were delivered at home. This finding is similar to a study conducted in Ethiopia and Nigeria, which found that health facility delivery increases the odds of VAS uptake (21, 32). The possible explanations might be that mothers who had ANC follow-up and health facility delivery may have received information, education, knowledge, and counseling from healthcare experts regarding the needs and importance of vitamin A supplementation (18, 21, 25). This underlines that strengthening pregnancy-related contact with health facilities is predicted to lead to increased usage of health services later on.

Regarding administrative zones, children aged living in the Western, Southern, and South West highlands were 2.05 times, 3.64 times, and 2.84 times more likely to be vitamin A supplemented than children living in Zanzibar. Meanwhile, the odds of vitamin A supplementation in children living in the South West Highlands were 44% less compared to children living in Zanzibar. The possible reasons might be that the regions with the highest prevalences of VAS were Njombe (91%) and Iringa (90%), which are both found in the southern highlands, while the regions with the lowest prevalences of VAS were Simiyu (19%) and Rukwa (20%), which are located in the South West highlands administrative zones. Further research is needed to understand the possible causes of variation among administrative zones.

### Limitations

Even though this study used a weighted pooled nationally representative TDHS survey in Tanzania with a sample size large enough to detect the true effect of the independent variables as well as the use of multilevel binary logistic regression analysis to achieve credible estimates and standard errors, the study cannot establish a causal association between vitamin A supplementation and independent variables due to the use of a cross-sectional study design. Furthermore, due to its reliance on respondents’ answers, the study might be affected by social desirability and recall biases.

## Conclusion

In general, the proportion of VAS among children in Tanzania is low compared to UNICEF’s target of 80%, with only one in two children taking vitamin A supplementation. This finding’s prevalence of VAS falls below the UNICEF 2022 report for Tanzania and Tanzania’s 2018 nutrition survey report, implying that the problem may be masked by recent reports of high coverage and that the situation is far from reality. Mother’s/caregiver’s working status, antenatal care, and place of delivery were positively associated individual-level factors with VAS. Besides, residing in the western, southern, and south-west highlands of Tanzania were found to be community-level factors associated with VAS. Therefore, interventions should be designed to improve the uptake of VAS among children whose mothers are currently not working. Provision and promotion of ANC and institutional delivery and strengthening of routine supplementation are recommended to increase coverage of childhood vitamin A supplementation, as ANC and institutional delivery were found to be positive factors affecting VAS. Moreover, special focus should be given to regions in the south-western highlands, and additional studies are necessary to investigate the root causes of regional variations in VAS.

## Data availability statement

Publicly available datasets were analyzed in this study. This data can be found here: https://dhsprogram.com.

## Ethics statement

The studies involving humans were approved by Medical Research Council of Tanzania and the Zanzibar Health Research Institute. The ICF’s Internal Review Board also reviewed the study. The studies were conducted in accordance with the local legislation and institutional requirements. Written informed consent for participation in this study was provided by the participants’ legal guardians/next of kin.

## Author contributions

AG: Conceptualization, Formal analysis, Software, Supervision, Writing – original draft, Writing – review & editing. YA: Formal analysis, Software, Writing – original draft, Writing – review & editing. BW: Formal analysis, Software, Writing – original draft, Writing – review & editing. GK: Formal analysis, Software, Writing – original draft, Writing – review & editing. KH: Formal analysis, Software, Writing – original draft, Writing – review & editing. SD: Formal analysis, Software, Writing – original draft, Writing – review & editing. AT: Formal analysis, Software, Writing – original draft, Writing – review & editing. AA: Formal analysis, Software, Writing – original draft, Writing – review & editing.

## Footnotes

^1^ https://dhsprogram.com/
